# Dynamic Holographic Display With Multiplexed Dielectric Metasurfaces

**DOI:** 10.1002/nap2.70114

**Published:** 2026-06-11

**Authors:** Yu Liu, Yu‐Tong Xiao, Xing‐Yuan Huo, Qing Cai, Rui Zhong, Ling‐Yuan Zhang, Ruwen Peng, Yongmin Liu, Mu Wang

**Affiliations:** ^1^ National Laboratory of Solid State Microstructures School of Physics Collaborative Innovation Center of Advanced Microstructures, and Jiangsu Physical Science Research Center Nanjing University Nanjing China; ^2^ Department of Mechanical and Industrial Engineering Northeastern University Boston Massachusetts USA; ^3^ Department of Electrical and Computer Engineering Northeastern University Boston Massachusetts USA

**Keywords:** dynamic display, metasurface holograms, multiplexed metasurfaces

## Abstract

With the increasing demand for tunable optical functionalities, metasurfaces capable of dynamic wave control have attracted growing attention. Compared with reconfigurable designs that rely on external stimuli, incident‐light‐modulated multiplexed metasurfaces offer a unique scalable pathway for achieving tunable functionalities. In this work, based on multiplexed metasurface schemes, we show hybrid multiplexing of polarization, wavelength, and orbital angular momentum (OAM) can be applied for dynamic tuning. We start to demonstrate a noise‐engineered metasurface platform that supports dynamic holographic display across multi‐nonorthogonal polarization channels, further combined with position, wavelength, and OAM multiplexing. Moreover, we extend this approach to achieve simultaneous multiplexing of polarization, wavelength, and OAM. This integration enables a single device to dynamically switch among holographic patterns under different incident light configurations, significantly enhancing the functional diversity of dynamic displays. Our strategy presents a highly compact and efficient solution for high‐capacity multi‐dimensional holography, with promising applications in advanced displays, data storage, optical encryption, and adaptive photonic systems.

## Graphical abstract

Based on multiplexed metasurfaces, we show hybrid multiplexing of polarization, wavelength, and orbital angular momentum can be applied for dynamic tuning. Our strategy presents a highly compact and efficient solution for high‐capacity, multi‐dimensional holography, with promising applications in advanced displays, data storage, optical encryption, and adaptive photonic systems.

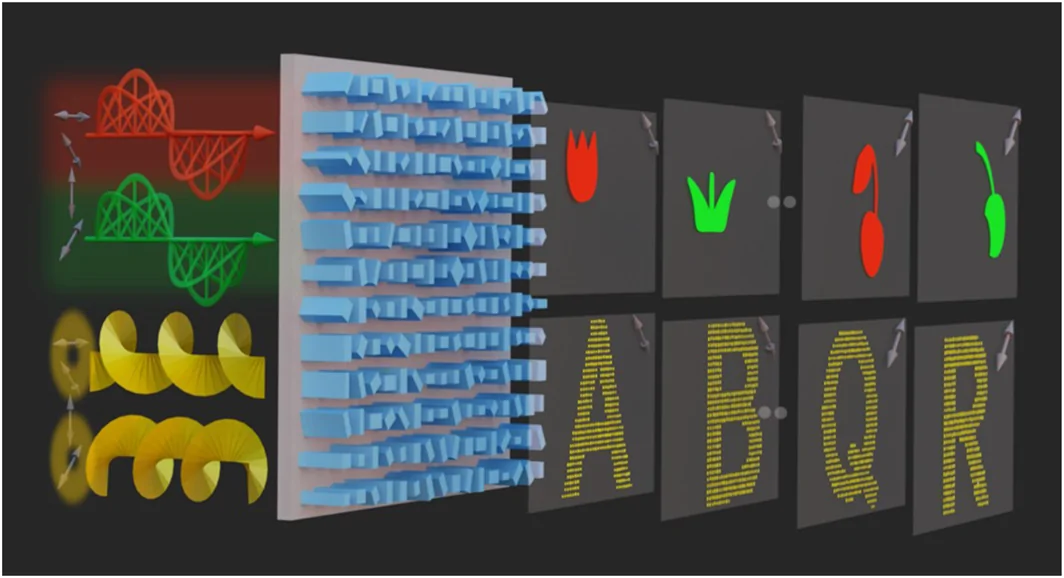

## 1 Introduction

Metasurfaces have emerged as a powerful platform for advanced optical manipulation, owing to their exceptional ability to control fundamental light properties, including phase [1, 2], amplitude [3, 4], polarization [5, 6, 7, 8], frequency [9, 10, 11], and orbital angular momentum (OAM) [12, 13]. However, for most metasurfaces, once fabricated, their optical functionalities are fixed. This intrinsic static nature severely limits their application in adaptive optical systems. Driven by the growing demand for tunable performance, research efforts have branched into two distinct paradigms for developing metasurfaces with dynamic capabilities. The first one leverages active materials or tunable mechanisms, such as phase transition [14, 15, 16, 17], liquid crystals [18, 19, 20], chemical reactions [21], electro‐optical tuning [22, 23], and mechanical strain [24, 25, 26, 27] to reconfigure metasurfaces dynamically. This allows tuning of the metasurface response by modifying nanostructure properties via various external stimuli. However, reconfigurable metasurfaces inevitably face difficulties in unconventional materials engineering and fabrication, whereas the need for external stimulus configurations often results in bulky systems. The other way is to combine fixed metasurfaces with dynamically modulated incident light, where the light itself acts as a tuning knob for the metasurface response. Thus far, a range of multiplexing strategies has been explored, incorporating polarization [28, 29, 30, 31, 32, 33, 34], wavelength [35, 36, 37, 38, 39, 40], and OAM [41, 42, 43, 44] multiplexing. These multiplexing approaches effectively encode several optical functions into a single passive device, offering a more scalable route for dynamic light control without the need for nanoscale reconfiguration.

Among these multiplexing schemes, polarization‐based control is particularly prominent due to the ease of generating and modulating the polarization of light. To further expand the capacity of polarization‐multiplexed dynamic displays, two primary strategies have been pursued: increasing the number of polarization channels and integrating polarization with other multiplexing techniques. However, the number of independent polarization channels is fundamentally limited by the degrees of freedom (DOFs) in the Jones matrix [28, 45], restricting single‐layer metasurfaces to a maximum of three channels. Recently, Xiong et al. demonstrated up to 11 independent holographic images using a single metasurface illuminated with different polarizations by introducing engineered noise, which breaks the limitation of polarization multiplexing [31]. A critical challenge persists, though, with cross‐talk growing more significant as the number of polarization channels increases. The second strategy integrates polarization multiplexing with other DOFs, such as position [46], OAM [47], and wavelength [39, 48, 49, 50]. Nevertheless, the majority of current schemes employ only two orthogonal polarization channels (e.g., orthogonal linear or circular polarizations). Therefore, the integration of multi‐channel polarization multiplexing technology with other DOFs is highly desirable, offering a promising solution for high‐capacity dynamic display based on multiplexed metasurfaces.

In this work, we propose a practical approach to integrate multi‐channel polarization multiplexing scheme based on noise engineering with other multiplexing techniques to enable dynamic holographic displays under incident light modulation. Our design involves a two‐step process: first, we extend polarization channels by calculating approximate solutions of the Jones matrix elements; then, we integrate the polarization multiplexing with other DOFs, and thereafter use a gradient descent algorithm to reduce crosstalk and enhance the signal‐to‐noise ratio of the images across all multiplexing channels. As a proof of concept, we experimentally demonstrate this polarization multiplexing scheme with multiple non‐orthogonal polarization channels. We further augment the multiplexing capability by combining this scheme with position, OAM, and wavelength multiplexing techniques, respectively. Moreover, we accomplish simultaneous multiplexing of polarization, wavelength, and OAM. This design strategy integrates the “limit‐breaking” polarization multiplexing scheme with other multiplexing mechanisms, significantly enhancing the multiplexing capability of metasurfaces. This work propels the development of dynamic high‐capacity multiplexing with metasurfaces, offering promising applications in the fields of colorful display, imaging, data storage, and information processing.

## 2 Design Principle

Figure 1 schematically illustrates the concept of dynamic holographic display via incident light modulation using a single‐layer dielectric metasurface. Composed of rectangular meta‐atoms with spatially varying lengths, widths, and rotation angles, the metasurface is designed to simultaneously support wavelength‐polarization and OAM‐polarization multiplexing. Specifically, it generates holographic patterns in the far field for wavelength‐polarization‐multiplexed illumination and holographic letters for OAM‐polarization‐multiplexed illumination. By changing the combination of polarization states with wavelength or OAM of the incident light, the holographic information displayed by this single device dynamically switches among multiple holographic images.

**FIGURE 1:**
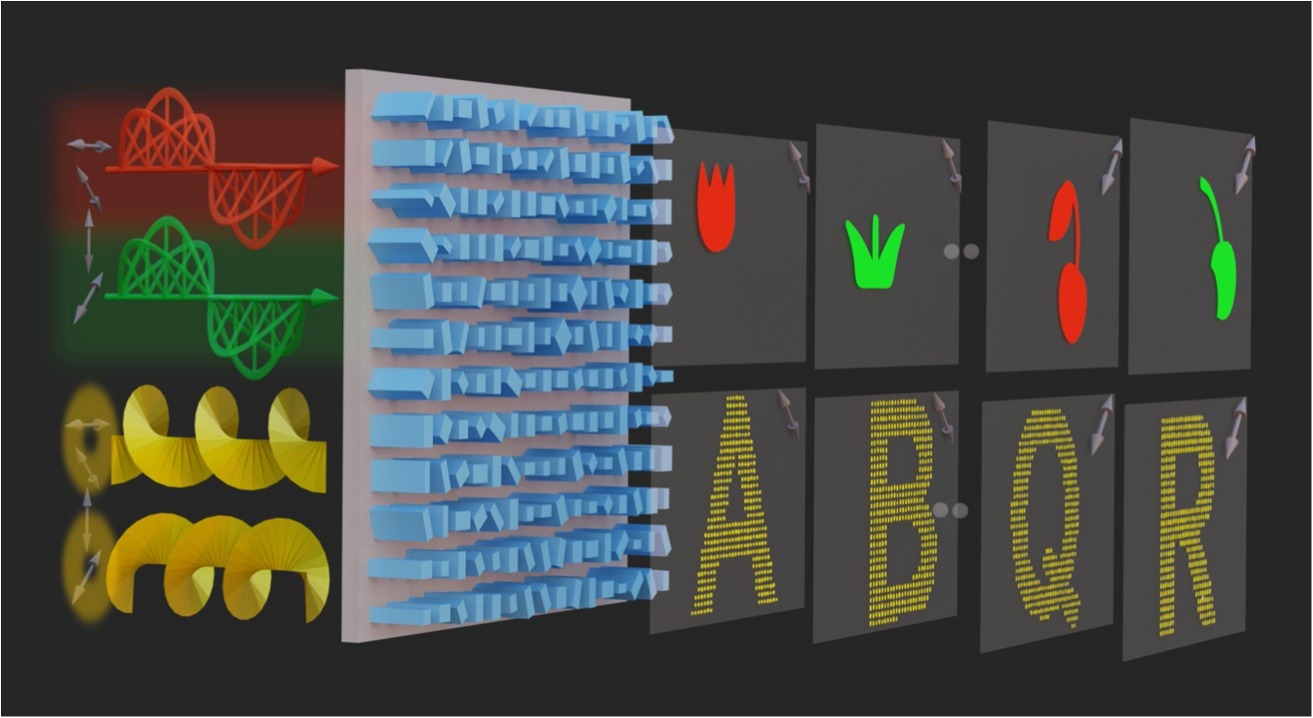
Schematic illustration of the dielectric metasurface for dynamic holographic display under incident light modulation. By changing the combination of polarization states with wavelength or orbital angular momentum (OAM) of the incident light, a single metasurface can generate different holographic images in the far field.

It is known that the Jones matrix of the unit cell in a metasurface (within the xy‐plane) can be expressed as $\left[\begin{array}{cc} J_{xx}\left(\lambda \right) & J_{xy}\left(\lambda \right)\\ J_{yx}\left(\lambda \right) & J_{yy}\left(\lambda \right) \end{array}\right]$, where the dispersive response of the meta‐atoms is considered. Due to the inherent mirror symmetry in the xy‐plane, the off‐diagonal elements are identical, meaning $J_{xy}\left(\lambda \right)=J_{yx}\left(\lambda \right)$. For simplicity, we consider the incident light of wavelength λ and complex amplitude $Ae^{i\phi }$ with the polarization state defined as ${\left[\begin{array}{cc} \cos\;\theta & \sin\;\theta \end{array}\right]}^{T}$, where θ denotes the polarization angle relative to the *x*‐axis. When detected by an analyzer oriented at the same angle, the output field immediately after the metasurface can be written as

(1) $$E^{n}\left(\theta ,\lambda ,\phi \right)=\left[\cos\;\theta \;\sin\;\theta \right]\left[\begin{array}{cc} J_{xx}\left(\lambda \right) & J_{xy}\left(\lambda \right)\\ J_{xy}\left(\lambda \right) & J_{yy}\left(\lambda \right) \end{array}\right]\left[\begin{array}{c} \cos\;\theta \\ \sin\;\theta \end{array}\right]Ae^{i\phi }.$$

Here, $E^{n}\left(\theta ,\lambda ,\phi \right)$ denotes the spatial distribution of the near field. Subsequently, the corresponding far‐field distribution at a propagation distance can be obtained via Fresnel diffraction theory:

(2) $$E^{f}\left(\theta ,\lambda ,\phi ,z\right)=E^{n}\left(\theta ,\lambda ,\phi \right)*H\left(z\right).$$

Here, $H\left(z\right)=\frac{\exp\left(ikz\right)}{i\lambda z}\exp\left[\frac{ik\left(x^{2}+y^{2}\right)}{2z}\right]$, k=2π/λ, and z denotes the propagation distance from the metasurface plane to the image plane. The symbol “∗” signifies the convolution operation over the spatial coordinates. Further, if we consider an incident field with uniform amplitude and a vortex phase profile, that is, $Ae^{i\phi }=\exp\left(il\varphi \right)$, where l is topological charge and φ is the azimuthal angle, and then extend the framework to incorporate the multiplexing of *m* polarization channels, the resulting far‐field distribution can be generalized as

(3) $$\left[\begin{array}{c} E^{f}\left(\theta_{1},\lambda ,l,z\right)\\ E^{f}\left(\theta_{2},\lambda ,l,z\right)\\ \begin{array}{c} \vdots \\ E^{f}\left(\theta_{m},\lambda ,l,z\right) \end{array} \end{array}\right]=\left\{\left[\begin{array}{ccc} \cos^{2}\theta_{1} & \sin\;2\theta_{1} & \sin^{2}\theta_{1}\\ \cos^{2}\theta_{2} & \sin\;2\theta_{2} & \sin^{2}\theta_{2}\\ & \vdots & \\ \cos^{2}\theta_{m} & \sin\;2\theta_{m} & \sin^{2}\theta_{m} \end{array}\right]\left[\begin{array}{c} J_{xx}\left(\lambda \right)\\ J_{xy}\left(\lambda \right)\\ J_{yy}\left(\lambda \right) \end{array}\right]e^{i\phi \left(l\right)}\right\}*H\left(z\right).$$

Here, the term in the braces is an m × 1 vector, and “∗” indicates the convolution of each element in this vector with H(z). As shown in Equation (3), we can establish the relationship between the image field and the feature of the incident light, including polarization state (θ), topological charge (*l*), wavelength (λ), and the propagation distance (*z*).

Based on Equation (3), we are able to perform hybrid multiplexing of polarization, wavelength, and OAM, together with position multiplexing as follows. (i) We start from polarization multiplexing. In order to achieve more than three independent channels, we employ approximate solutions (e.g., the least‐squares solutions) of the Jones matrix elements with sufficient engineered noise [31], thereby opening new polarization channels. (ii) Combining polarization multiplexing with position multiplexing. The two degrees of freedom, that is, polarization and position, are independent. Thus, position multiplexing can be applied within each polarization channel, and the aforementioned polarization multiplexing framework is subsequently applied, enabling joint polarization and position multiplexing. Similar approach is employed for multiplexing of polarization and OAM. For polarization and wavelength multiplexing, we implement the polarization multiplexing scheme separately at different wavelengths. (iii) Further by extending the approach in (ii), we can achieve simultaneous multiplexing of polarization with both wavelength and OAM. With such hybrid multiplexing, multiple channels can be dynamically opened for optical holography by modulating the incident light or changing the display plane.

However, there exists the crosstalk among different multiplexing channels. To mitigate this crosstalk and enhance the signal‐to‐noise ratio of the holographic images, we employ a gradient descent optimization algorithm to globally optimize the results across all channels. According to Equation (3), the far‐field distributions for all aforementioned multiplexing configurations can be computed by assigning desired combinations of parameters. The Jones matrix elements obtained from the least‐squares method serve as the initial solution. By deriving the gradient of the loss function with respect to the Jones matrix elements, we iteratively optimize the design until the desired performance is achieved. In this work, the optimization runs for up to 200 iterations with a step size of 0.01, stopping when the relative change in the loss function falls below 1 × 10^−3^. Throughout the process, the absolute values of the Jones matrix elements are constrained not to exceed unit. With this approach, we can achieve an optimized design of the metasurface.

In the metasurface design, we select the unit cell consisting of two pairs of amorphous silicon (α‐Si) nanopillars on an indium tin oxide (ITO) coated glass substrate. These nanopillars are characterized by three independently tunable geometric parameters: length (*L*), width (*W*), and rotation angle (*ϕ*), which collectively enable precise control over the incident light. The α‐Si nanopillars are designed to have an identical height of *H* = 600 nm with a lattice period of *P* = 360 nm. The length *L* and width *W* of the rectangular nanopillar vary from 60 to 280 nm. For the required Jones matrix elements of each pixel on the metasurface, we use a modified genetic algorithm to search for proper geometric parameters (*L*, *W*, *ϕ*) of two pairs of nanopillars based on the simulated amplitude and phase databases obtained by full‐wave simulation with the finite‐difference time domain (FDTD) method.

## 3 Results

### 3.1 Multi‐Channel Polarization Multiplexing

To experimentally validate this concept, we fabricated the metasurface and demonstrated the results of five‐channel polarization multiplexing. Figure 2a displays the optical and scanning electron microscopy (SEM) micrographs of the sample, which measures 200 μm × 200 μm. The sample was fabricated using standard electron beam lithography (EBL) followed by inductively coupled plasma‐reactive ion etching (ICP‐RIE). The details of the fabrication procedure are outlined in the Methods. The working wavelength of the metasurface is 800 nm. Figure 2b presents the simulation results, in which five independent holographic images (“A”, “B”, “C”, “D”, and “E”) can be generated at the corresponding polarization states (0°, 36°, 72°, 108°, 144°). The measured results are presented in Figure 2c, which closely match the simulation results. To assess cross‐talk between images at distinct channels, we employ the correlation coefficients matrix as a quantitative metric [31], where each element $\rho_{m,n}$ characterizes the correlation between the m‐th target image and the n‐th reconstructed image. The element $\rho_{m,n}$ is mathematically expressed as

(4) $$\rho_{m,n}=\frac{\mathrm{Cov}\left(T_{m},R_{n}\right)}{\sqrt{\mathrm{Var}\left(T_{m}\right)\cdot \text{Var}\left(R_{n}\right)}}.$$

**FIGURE 2:**
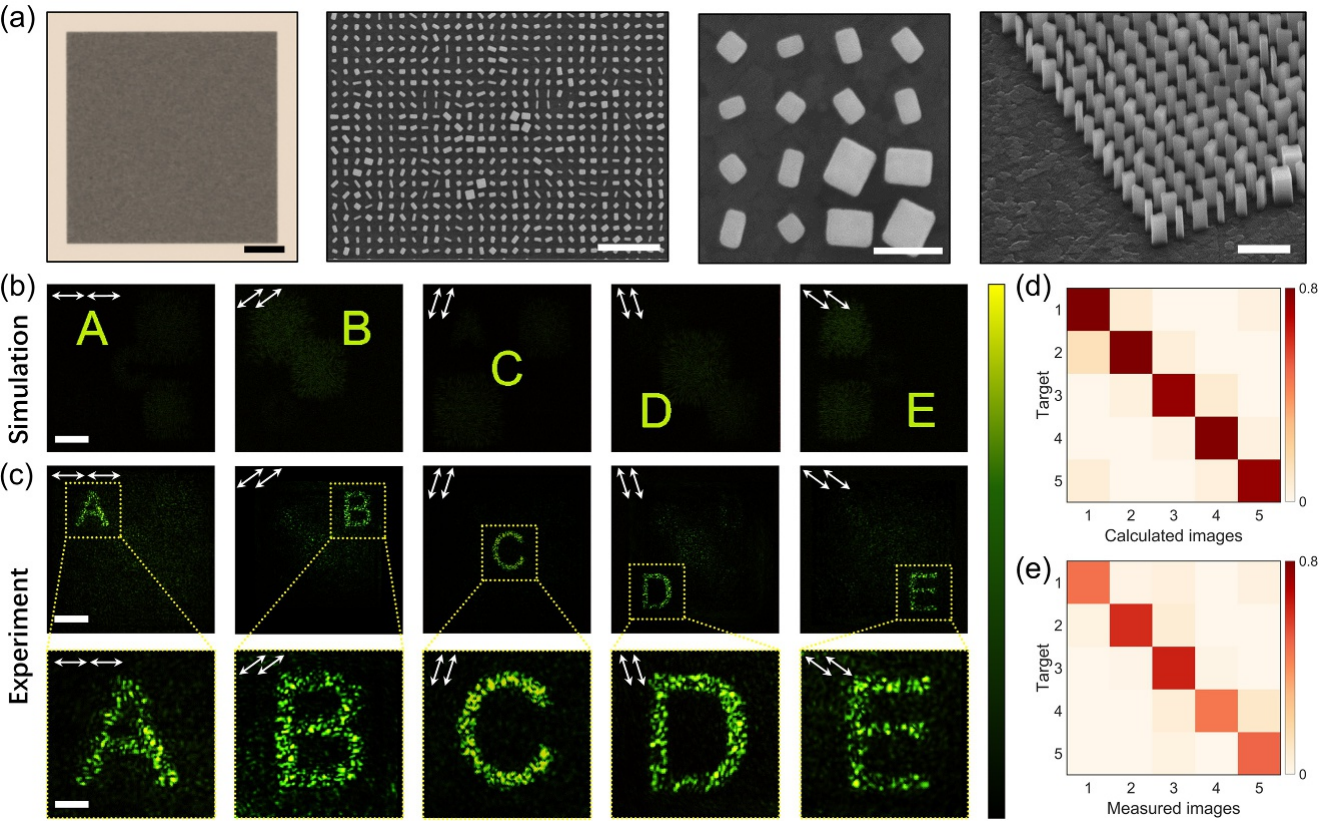
Simulation and experimental demonstration of the dielectric metasurface for five‐channel polarization multiplexing. (a) Optical and SEM micrographs of the fabricated sample with different magnifications. The scale bars from left to right are 50 μm, 2 μm, 400 nm, and 800 nm, respectively. (b) Simulated and (c) measured holographic images with different polarization incidences/analyzations, which are marked by the white arrows in the upper left. When the incident light is polarized along 0°, 36°, 72°, 108°, and 144°, the metasurface can generate five independent holographic images, including letters “A”, “B”, “C”, “D”, and “E”. The scale bar in (b) is 40 μm. The scale bars in (c) are 40 μm (top) and 13 μm (bottom), respectively. (d) Simulated and (e) measured correlation coefficients matrix between the target images and holographic images. Here, 2.00 mW laser power and 10 ms exposure time are applied.

Here, $T_{m}$ and $R_{n}$ stand for the intensity distributions of the m‐th target and n‐th reconstructed images, respectively. $\text{Cov}\left(T_{m},R_{n}\right)$ represents the covariance between $T_{m}$ and $R_{n}$, while $\text{Var}\left(T_{m}\right)$ and $\text{Var}\left(R_{n}\right)$ denote the variances of $T_{m}$ and $R_{n}$. For five‐channel polarization multiplexing, we computed the corresponding 5 × 5 correlation coefficients matrix: the diagonal element $\rho_{m,m}$ indicates the signal strength of the m‐th channel, and off‐diagonal elements $\rho_{m,n}$ (m≠n) reflect the cross‐talk between the m‐th channel and the n‐th channel. Figure 2d,e present the calculated and measured correlation matrix. The off‐diagonal terms are all below 0.2, confirming that these polarization channels are relatively uncorrelated and hence independent. As the number of channels increases, the correlation between different channels becomes stronger, leading to higher crosstalk and lower image fidelity. By adjusting the design parameters and relieving the threshold conditions [31], the number of the feasible polarization channels in this scheme can be increased significantly.

### 3.2 Position and Polarization Multiplexing

To demonstrate the scalability of the polarization multiplexing scheme based on noise engineering, we further integrate it with multiplexing schemes of other degrees of freedom, including position, wavelength, and OAM. First, we realized position and polarization multiplexing. Here, we employ the previous set of five polarization channels (0°, 36°, 72°, 108°, 144°), and each channel is assigned two different holographic images at the planes 200 and 400 μm away from the metasurface. The target holographic images are designed as 10 different zodiac signs. The metasurface is 200 μm × 200 μm in size, consisting of 276 × 276 pixels. For each polarization channel, a 3D multiplane holography algorithm [51] is employed to obtain the required phase profile, which could generate two holographic images at different target planes. Subsequently, the phase distributions required for all polarization channels are substituted into the polarization multiplexing scheme for numerical solution. Based on the aforementioned method, the desired α‐Si metasurface for position and polarization multiplexing is obtained. Figure 3a,b show the simulated and measured results of 10 different zodiac signs under different polarization channels at the planes 200 and 400 μm. Each channel displays the designed holographic image. Notably, the scalar Fresnel diffraction model used in our simulation neglects vectorial near‐field effects of the meta‐atoms, which can partially account for the increased speckle‐like noise observed in the experiment. Figure 3c,d present the calculated and measured correlation matrix. As indicated by the diagonal elements of the measured correlation matrix, the dominant energy is confined to the target channels; however, the energy distribution across channels remains uneven. This imbalance could be alleviated by an optimization strategy that dynamically adjusts channel weights according to their respective loss values, so as to balance the energy among channels. To assess the effectiveness of the proposed optimization, we calculate the structural similarity index measure (SSIM) and normalized cross correlation (NCC) [49] of the simulated images before and after inter‐channel energy balancing, as shown in Figure 3e,f. The results indicate that the image quality for most channels exhibits a clear enhancement after optimization.

**FIGURE 3:**
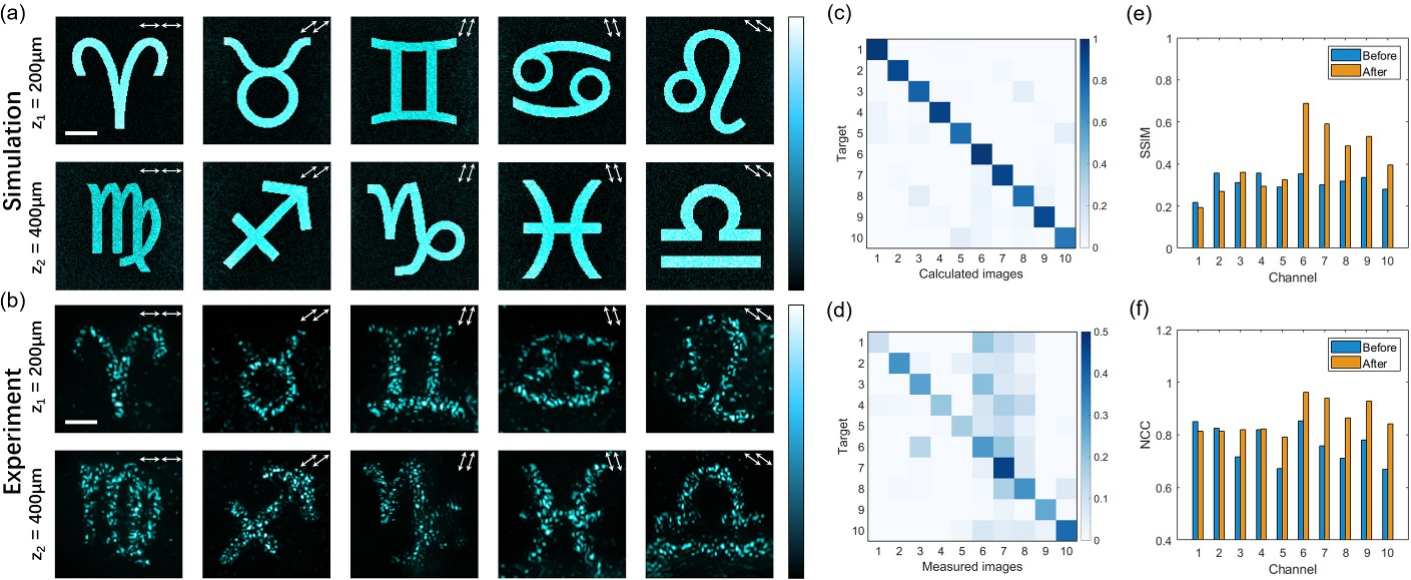
Simulation and experimental demonstration of polarization and position multiplexing. (a) Simulated and (b) measured holographic images of 10 zodiac signs at different z planes with different polarization incidences/analyzations. Five linear polarization channels (0°, 36°, 72°, 108°,144°) are chosen, and two different holographic images can be generated simultaneously at the planes 200 and 400 μm away from the metasurface for each polarization channel. The white arrows in the upper right indicate the orientation of the polarizer and analyzer. The scale bars in both (a) and (b) are 13 μm. (c) Simulated and (d) measured correlation coefficients matrix between the target images and holographic images. (e) SSIM and (f) NCC before and after inter‐channel energy balancing optimization. Here, 2.00 mW laser power and 10 ms exposure time are applied.

### 3.3 OAM and Polarization Multiplexing

Furthermore, we integrate the polarization multiplexing scheme based on noise engineering with OAM multiplexing. Owing to the unbounded nature of helical modes and their intrinsic orthogonality, OAM‐multiplexing holography shows unprecedented capacity for optical information encoding. Here, the linear polarization channels are added to the OAM‐multiplexed holography, with which the information capacity is further enhanced, enabling dynamic multi‐channel holographic displays by controlling the combination of topological charges and polarization states of the incident light. The multiplexing approach is schematically illustrated in Figure 4a. We select five linear polarization states (0°, 36°, 72°, 108°, 144°), with each polarization channel combined with three OAM modes (*l* = −3, 0, 3), resulting in a total of 15 independent channels. The target images consist of 15 alphabet letters spatially separated in the xy‐plane. To avoid overlap of OAM signals in momentum space, the images are first sampled using a 2D Dirac comb. The required phase distributions for the sampled holographic images are calculated using the Gerchberg‐Saxton (GS) algorithm and subsequently superposed with spiral phase profiles corresponding to the desired OAM states, yielding OAM‐selective holograms. Only the given OAM mode with an inverse topological charge (−*l*) can recover the fundamental Gaussian mode with a solid‐spot intensity distribution in each pixel at the target plane, and hence to distinctively reconstruct the holographic image. For each polarization channel, three OAM‐selective holograms with different topological charges (*l* = 3, 0, −3) are superposed to form the OAM‐multiplexed hologram. The resulting superposed optical field serves as the near‐field distribution for each polarization channel. Finally, based on the aforementioned polarization multiplexing framework, we designed and realized an α‐Si metasurface to achieve simultaneous OAM and polarization multiplexing.

**FIGURE 4:**
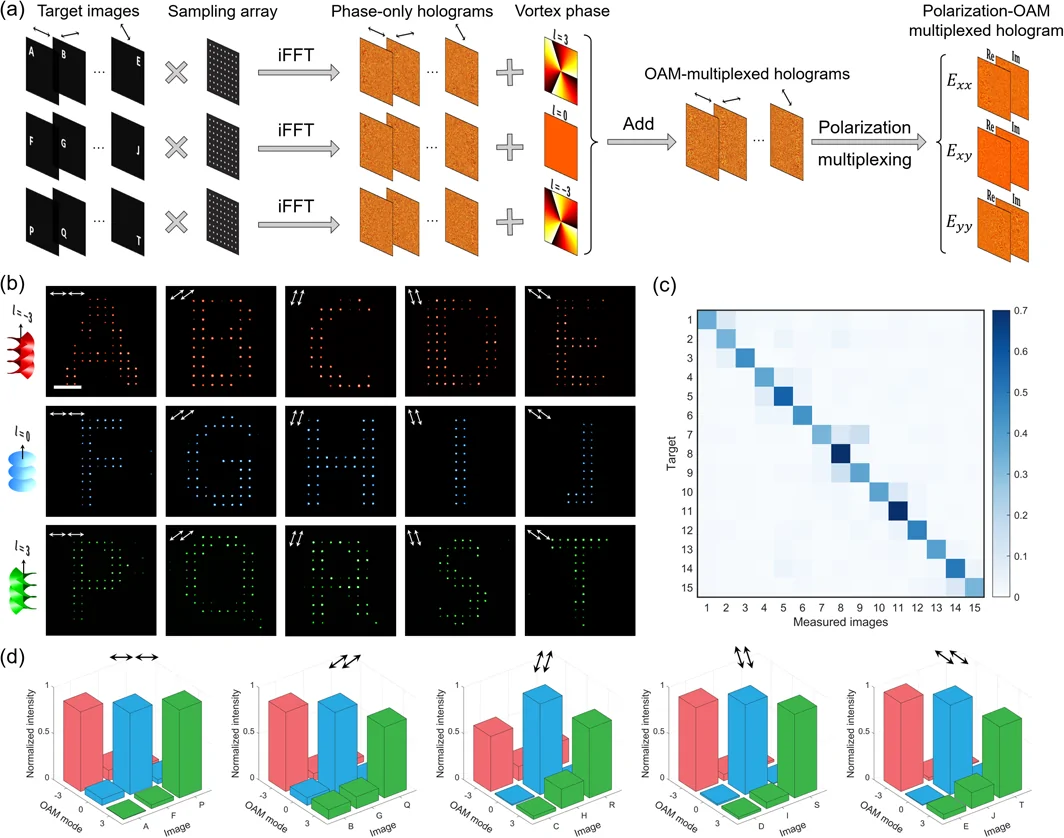
Design and experimental demonstration of polarization and OAM multiplexing. (a) Design strategy of the polarization‐OAM multiplexed hologram. (b) Experimentally measured 15 different holographic images when illuminated by OAM beams with different topological charges (*l* = −3, 0, 3) and polarization states (0°, 36°, 72°, 108°,144°). The white arrows in the upper left indicate the orientation of the polarizer and analyzer. The color indicates the topological charge of the incident vortex beam (red: *l* = −3; blue: *l* = 0; green: *l* = 3). The scale bar is 13 μm. (c) Measured correlation coefficients matrix between the target images and holographic images. (d) Analysis of OAM modal crosstalk for the reconstructed holograms under different polarization channels (left to right: 0°, 36°, 72°, 108°, and 144°). Here, 2.00 mW laser power and 10 ms exposure time are applied.

To experimentally validate the design, we fabricated the metasurface with the size of 250 μm × 250 μm, consisting of 344 × 344 pixels. The measured results are illustrated in Figure 4b. The different colors of the images represent the topological charges of the incident OAM beams, whereas the directions of the arrows above the images represent the incident and analyzed polarizations. The target images are well reconstructed as distinct Gaussian spots only when illuminated by OAM beams with designed topological charge and polarization state. Otherwise, the reconstructed signals exhibit doughnut‐shaped intensity distributions. To further improve the OAM selectivity, a fundamental mode filtering aperture array was added to the measured results to rule out the high‐order OAM modes with doughnut‐shaped intensity distributions. Figure 4c demonstrates the measured correlation matrix. The off‐diagonal elements of the matrix are less than 0.16, indicating the channels are relatively independent. Further, to quantitatively evaluate the OAM selectivity [43, 52], we measured intensities of the reconstructed holograms for different OAM modes across various polarization channels, as shown in Figure 4d. The target channels exhibit an average normalized intensity of 87%, demonstrating very effective OAM selectivity.

### 3.4 Wavelength and Polarization Multiplexing

As another demonstration, we combine wavelength and polarization multiplexing to achieve dynamic holographic display. First, we obtain the required phase distributions for wavelength multiplexing under each polarization channel. Since optical responses of nanostructures are wavelength‐correlated, directly computing target phase distributions independently for each wavelength channel makes it fundamentally challenging to identify meta‐atoms satisfying all spectral requirements simultaneously. To address this constraint, we propose a modified gradient descent algorithm. Actually for the propagation phase modulated by dielectric structural units with heights comparable to the wavelength, the phase response of these units can be expressed as $\phi \left(\lambda \right)=\frac{2\pi }{\lambda }n_{eff}\left(\lambda \right)\cdot D$, where $n_{eff}$ and D denote the effective refractive index and thickness of the structural unit, respectively [53, 54, 55]. Under certain operating bandwidth, the variation of $n_{eff}\left(\lambda \right)$ with wavelength can be neglected, then the optical phase response of the structure exhibits an approximately linear relationship with 1/λ, so we can express the phase at wavelength $\lambda_{n}$ as $\varphi \left(\lambda_{n}\right)\approx \varphi \left(\lambda_{1}\right)+k\left(1/\lambda_{n}-1/\lambda_{1}\right)$. By incorporating this linear dispersion relation, we co‐optimize the phase profile $\varphi \left(\lambda_{1}\right)$ at the reference wavelength $\lambda_{1}$ and the phase dispersion coefficient k across the range $\left\{1/\lambda_{n},1/\lambda_{1}\right\}$, thereby obtaining the phase distributions that the metasurface needs to satisfy at different wavelengths. Compared with applying the GS algorithm separately at each wavelength to obtain the phase distributions, this strategy makes it easier to find structures that simultaneously satisfy the target phase profiles for all wavelengths.

Based on the aforementioned polarization and wavelength multiplexing method, the required dispersion Jones matrix elements ($J_{xx}\left(\lambda \right),J_{xy}\left(\lambda \right),J_{yy}\left(\lambda \right)$) of the structures can be derived. We then employ the FDTD simulations to obtain the phase and amplitude of meta‐atoms across the operational wavelengths to constitute a complex‐amplitude database. A modified genetic algorithm is subsequently used to identify nanostructures that match the target dispersive Jones matrix.

To validate the proposed method, we employ five linear polarization channels (0°, 36°, 72°, 108°, 144°) multiplexed with two wavelengths (700 and 850 nm). Across this wavelength range, the phase varies approximately linearly with 1/λ. This design develops a meta‐device that achieves dynamic ten‐channel holographic display by modulating the combination of the wavelength and polarization state of the illumination light. The fabricated metasurface is 200 μm × 200 μm in size. The simulated results are shown in Figure 5a. Under 700 nm illumination with polarization directions varying from 0° to 144°, the metasurface sequentially reconstructs the letters “W”, “O”, “R”, “L”, and “D”. Switching to 850 nm illumination with the same polarization series changes the output to “M”, “E”, “T”, “A”, and a heart symbol. The experimental results in Figure 5b show good agreement with simulations, though minor background noise is observed at 700 nm due to optical loss in α‐silicon nanopillars at this wavelength, which slightly degrades image quality. By employing low‐loss dielectric materials, we can further extend this design to other visible wavelengths. For instance, titanium dioxide (TiO_2_) or silicon nitride (Si_3_N_4_) with higher transmission amplitude across the visible to near‐infrared spectrum are expected to enhance both holographic efficiency and image fidelity at the target wavelength.

**FIGURE 5:**
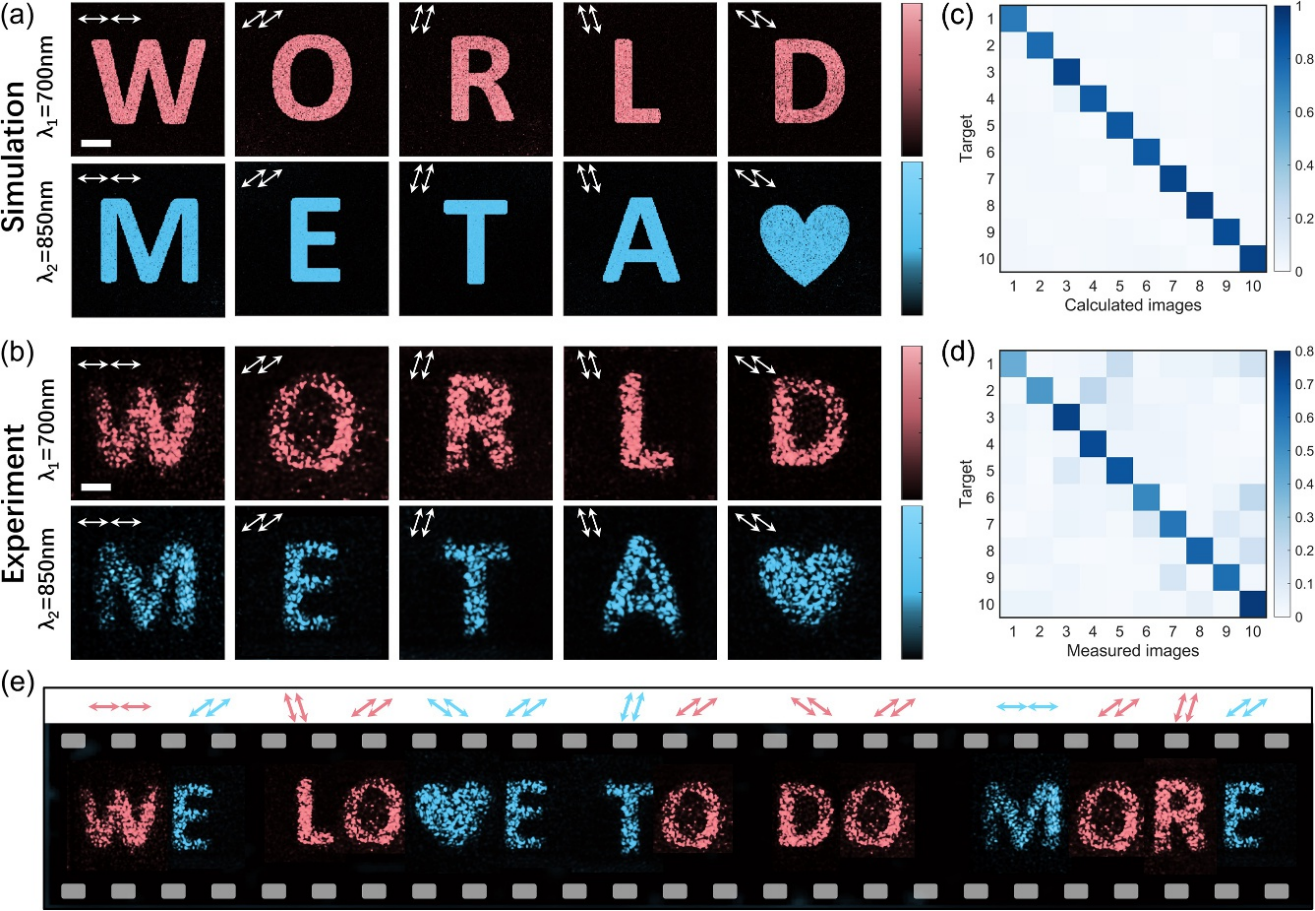
Simulation and experimental demonstration of polarization and wavelength multiplexing. (a) Simulated and (b) measured holographic images under the illumination of light with different wavelengths and polarization states. The metasurface can generate different characters forming the phrase “WORLD META♥” by switching the polarization channel from 0° to 144° at *λ*_1_ = 700 nm and *λ*_2_ = 850 nm, respectively. The white arrows in the upper left indicate the orientation of the polarizer and analyzer. The color indicates the wavelength of the incident light (pink: 700 nm; blue: 800 nm). The scale bars in both (a) and (b) are 13 μm. (c) Simulated and (d) measured correlation coefficients matrix between the target images and holographic images. (e) By modulating the state of incident light in a different sequence, the metasurface dynamically displays an alternative phrase. Arrows above the images indicate the polarization direction and the wavelength of incident light. Here, 0.49 mW laser power and 10 ms exposure time are applied at *λ*_1_ = 700 nm; whereas 2.13 mW laser power and 30 ms exposure time at *λ*_2_ = 850 nm.

Figure 5c,d present the simulated and measured correlation coefficients matrices between target and reconstructed images. The simulated matrix exhibits diagonal elements > 0.7 and off‐diagonal elements < 0.1, indicating low crosstalk between different channels. The experimental results show slightly degraded performance compared with simulations, but the primary energy remains concentrated along the main diagonal, with discrepancies attributable to fabrication imperfections.

To further evaluate the device performance, we measured the efficiency and signal‐to‐noise ratio for all the channels. The efficiency at each channel is defined as the ratio of total intensity at the image plane to total incident energy received by the metasurface area under the corresponding wavelength and polarization state [33, 56]. It is found that at 700 nm, the efficiencies across the five polarization channels range from 10.2% to 12.3% per channel; whereas at 850 nm, the efficiencies range from 14.7% to 16.1% per channel, all of which are comparable or even higher than those in Ref.33. Concurrently, the signal‐to‐noise ratio (SNR) within the region of interest is quantified to evaluate image fidelity, defined as the ratio of the peak intensity to the standard deviation of the background noise [57, 58]. The average SNR across the five channels is 26.8 at 700 nm and 42.5 at 850 nm. These results confirm that the metasurface achieves not only efficient energy distribution into multiple holographic channels but also high‐fidelity information retrieval. Better performance can be achieved by further reducing the imperfections in sample fabrication, which may introduce speckle‐like noise into the reconstructed holograms.

In addition, we demonstrate the dynamic holographic display capabilities of this multiplexed metasurface. By changing the polarization channel from 0° to 144° at *λ*_1_ = 700 nm and *λ*_2_ = 850 nm, the metasurface progressively displays the phrase “WORLD ♥ META” (Video S1). As shown in Figure 5e, altering the illumination sequence allows the device to generate another message “WE LOVE TO DO MORE”. Arrows above the images indicate the polarization direction and the wavelength of incident light.

### 3.5 Simultaneous Multiplexing of Polarization, Wavelength, and OAM

Finally, to further expand the multiplexing capabilities of the metasurface, we propose a hybrid multiplexing scheme that enables simultaneous wavelength‐polarization multiplexing and OAM‐polarization multiplexing. The system integrates a total of 15 multiplexing channels within a single metasurface, including: (i) three polarization states (0°, 60°, 120°) multiplexed with two wavelengths (700 nm, 800 nm) and (ii) three polarization states (30°, 90°, 150°) multiplexed with three OAM modes (*l* = −3, 0, 3). The experimental setup for hologram characterization is schematically illustrated in Figure 6a (See more details in Methods). Under varying illumination conditions, including polarization angle, wavelength, and OAM mode, a single metasurface reconstructs distinct holographic images in the far field, as shown in Figure 6b. The wavelength‐polarization multiplexed channels are designed to display patterned holographic images. When the polarization channel is set at 0°, the sun and its radiating light patterns are reconstructed under the illumination of 700 and 800 nm, respectively. While changing the polarization directions to 60° with the same wavelength, the metasurface generates the cloud and raindrop patterns, composing the image of rain. At 120° polarization channel, stellar and lunar patterns are produced, composing the image of night. The OAM‐polarization multiplexed channels are utilized to display holographic letters. Under the illumination of light with different OAM modes and polarization states, nine specific letters are clearly reconstructed. Thus, by modulating the incident light parameters—wavelength, polarization, and OAM—a single metasurface enables dynamic holographic display across dual‐information channels, as demonstrated in Figure 6c. The first row serves as the image channel displaying various natural patterns, whereas the second row functions as the character channel providing textual explanations for the corresponding patterns. The arrow in the gray area above the images in the first row denotes the polarization direction and wavelength of the incident light for the wavelength‐polarization channel. Meanwhile, the polarization direction and topological charge of the incident light for the polarization‐OAM multiplexing channel are indicated by the corresponding arrow and number positioned beneath the letters in the second row.

**FIGURE 6:**
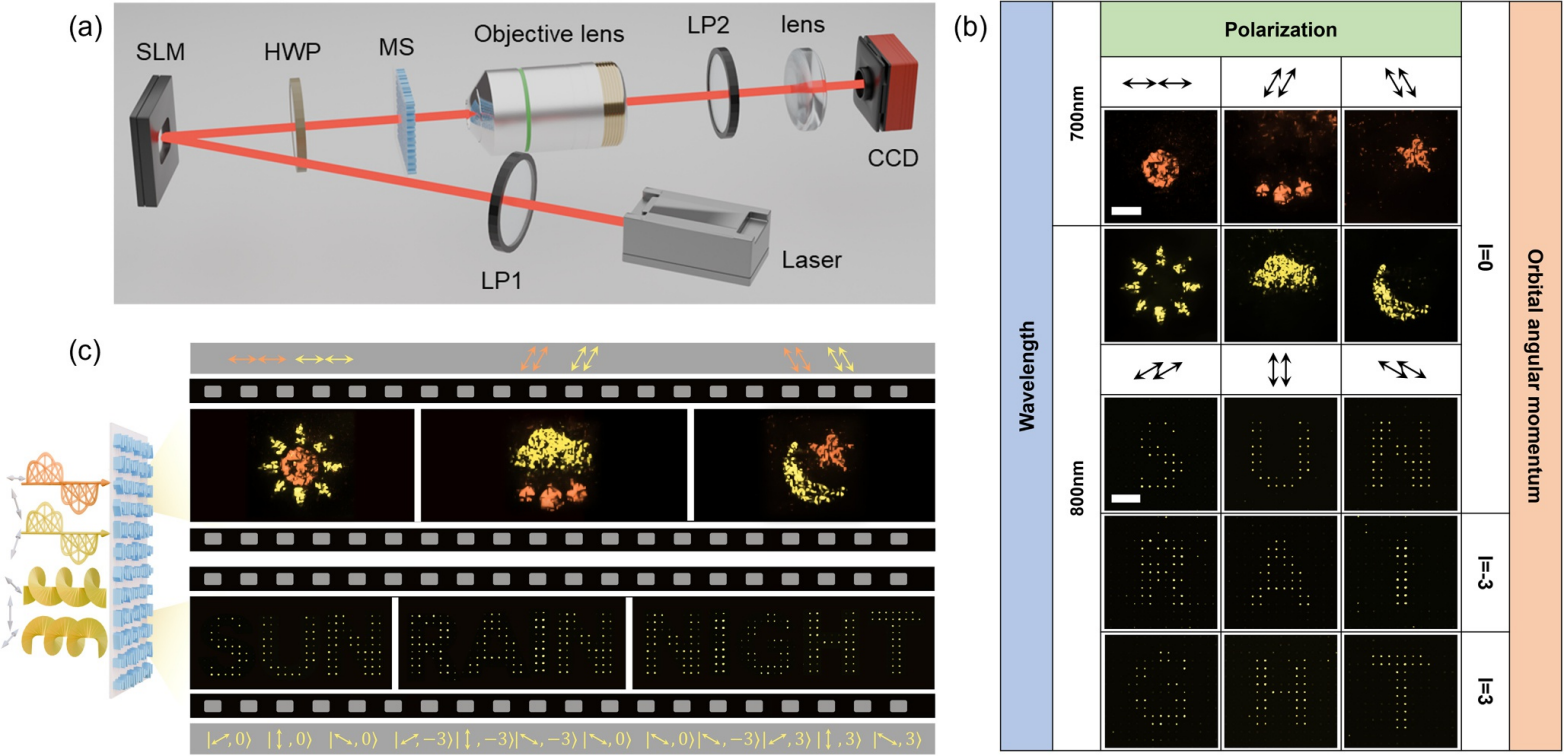
Experimental demonstration of the dielectric metasurface for simultaneous polarization‐OAM and polarization‐wavelength multiplexing. (a) Schematic of the optical setup. (b) Experimentally measured holographic images with different incident conditions (polarization, wavelength, and OAM) on a single metasurface. Three polarization channels (0°, 60°, 120°) are multiplexed with wavelength (*λ*_1_ = 700 nm and *λ*_2_ = 800 nm), and another three (30°, 90°, 150°) are multiplexed with OAM (*l* = −3, 0, 3). The arrows indicate the orientation of the polarizer and analyzer, and the arrow color indicates the wavelength of the incident light (orange: 700 nm; yellow: 800 nm). The scale bars in (b) are 20 μm (top) and 13 μm (bottom). (c) Under different multiplexing strategies, a dual‐channel dynamic display is achieved by a single metasurface. The labels in the gray areas at the top and bottom of the images represent the incident states. Here, 0.49 mW laser power and 10 ms exposure time are applied at 700 nm; whereas 2.00 mW laser power and 20 ms exposure time at 800 nm. CCD: charge‐coupled device, HWP: half‐wave plate, LP: linear polarizer, MS: metasurface, SLM: spatial light modulator.

## 4 Conclusion

We have demonstrated the polarization multiplexing scheme based on noise engineering, and integrated this approach with multiple optical degrees of freedom, including position, wavelength, and orbital angular momentum, thus successfully attaining multi‐channel dynamic holographic display under the modulation of incident light by single‐layer metasurfaces. Specifically, the proposed scheme is not limited to multiplexing with only one degree of freedom. We have further accomplished the simultaneous multiplexing of polarization with two other degrees of freedom (wavelength and OAM). When subjected to different incident conditions, the system is capable of dynamically showing image information and textual information. As a result, the functional diversity of dynamic display is remarkably enhanced.

In addition, we can integrate the current design scheme with liquid crystals to achieve polarization switching by changing the electrical voltage. Moreover, by using software to control tunable laser sources for wavelength adjustment and manipulating spatial light modulators to alter the applied spiral phase distribution, we can realize electrically controlled fast‐switching dynamic holographic display. By enhancing the multiplexing capability of metasurfaces, we have provided a new approach for future miniaturized and multifunctional dynamic displays. The approach holds great promise for applications in colorful displays, imaging, data storage, advanced information processing, and other fields with dynamic or multifunctional light manipulation.

## 5 Methods

### 5.1 Sample Fabrication

The metasurface devices were fabricated as arrays of α‐Si nanopillars on indium tin oxide (ITO) coating glass substrate. The fabrication began with the deposition of a 600 nm thick α‐Si film via plasma‐enhanced chemical vapor deposition (PECVD). A layer of polymethyl methacrylate (PMMA) was then spin‐coated onto the silicon surface and baked at 180°C for 90 s. Subsequently, the pattern of the metasurface was defined by electron beam lithography. An aluminum oxide (Al_2_O_3_) layer was deposited by electron beam evaporation, which served as a hard mask after the lift‐off process. The pattern was then transferred to the silicon layer using inductively coupled plasma ‐ reactive ion etching (ICP‐RIE) with an optimized gas mixture (SF_6_/C_4_F_8_). Finally, the Al_2_O_3_ mask was removed with ammonium hydroxide to reveal the complete α‐Si nanopillar structures. The fabricated metasurface devices typically possess dimensions of 200 and 250 μm.

### 5.2 Experimental Setup

A laser beam with tunable wavelengths (700 nm/800 nm/850 nm) is generated with a supercontinuum laser source. The beam passes through a linear polarizer (LP1) with horizontal polarization and then is projected onto the spatial light modulator (SLM). Then the beam is modulated and reflected by the SLM, yielding the desired spiral wavefronts. A half‐wave plate (HWP) is placed after the SLM to control the polarization of the incident light beam. The transmissive field generated by the metasurface is collected by an objective lens with a magnification of 20×, and subsequently analyzed by another linear polarizer (LP2). The holographic images are recorded by a charge‐coupled device (CCD) camera.

## Author Contributions

Y.L., Y.‐T.X., and X.‐Y.H. contributed equally to this work. R.P., Y.L., and Y.‐T.X. conceived this work. Y.L. and Y.‐T.X. contributed to the theoretical design. Y.L., Y.‐T.X., and X.‐Y.H. fabricated the samples with the assistance of Q.C. and L.‐Y.Z.; Y.L., Y.‐T.X., X.‐Y.H., and R.Z. performed the optical experiments. Y.L., Y.‐T.X., X.‐Y.H., and R.P. contributed to the data analysis and discussion. R.P., Y.M.L., and M.W. directed and supervised the research. Y.‐T.X., X.‐Y.H., R.P., Y.M.L., and M.W. drafted and finalized the manuscript. All authors revised the manuscript.

## Funding

Ruwen Peng and Mu Wang acknowledge the support of the National Key R&D Program of China (Grants 2022YFA1404303 and 2020YFA0211300), the National Natural Science Foundation of China (Grants 12234010, 61975078, and 11974177), and the Natural Science Foundation of Jiangsu Province (Grant BK20233001).

## Conflicts of Interest

The authors declare no conflicts of interest.

## Supporting information


**Video S1:** The experimentally dynamic display of holographic images with a multiplexed metasurface when changing the polarization and wavelength of incident light.

## Data Availability

The data that support the findings of this study are available from the corresponding author upon reasonable request.
